# A Study of Clinico-Etiological Profiles and Outcomes of Pleural Effusion in a Tertiary Care Hospital

**DOI:** 10.7759/cureus.115293

**Published:** 2026-08-27

**Authors:** Kabotoli Jakhalu, Ritu Rakholia, Prerna Chamoli, Mohd Maroof

**Affiliations:** 1 Paediatrics, Government Medical College, Haldwani, IND; 2 Community Medicine, Rani Durgavati Medical College, Banda, IND

**Keywords:** adenosine deaminase, children, parapneumonic effusion, pleural effusion, tubercular pleural effusion

## Abstract

Background: Pleural effusion in children is an important cause of respiratory morbidity with diverse infective and non-infective etiologies. Clinical presentation, biochemical profile, microbiological findings, and imaging characteristics play a major role in determining etiology, severity, treatment strategy, and overall outcome in pediatric pleural effusion.

Objectives: The study aimed to evaluate the clinico-etiological profile and outcomes of pleural effusion in children admitted to a tertiary care hospital.

Methods: This cross-sectional study was conducted in the pediatric ICU and pediatric ward of Dr. Susheela Tiwari Hospital, Government Medical College, Haldwani, India. Eighty children with pleural effusion were enrolled. Clinical examination, hematological investigations, pleural fluid analysis, adenosine deaminase (ADA) estimation, cartridge-based nucleic acid amplification test (CBNAAT), microbiological studies, chest radiography, and ultrasonography were performed.

Results: Tubercular pleural effusion was the commonest etiology (36, 45.0%), followed by parapneumonic effusion (18, 22.5%) and empyema thoracis (11, 13.75%). Fever (62, 77.5%), cough (68, 85.0%), and respiratory distress (54, 67.5%) were predominant symptoms. Exudative effusion was observed in 68 (85.0%) patients. Pleural ADA was significantly higher in tubercular effusion (84 ± 22 IU/L; p < 0.001). Intercostal drain (ICD) insertion, ultrasonographic loculation, and tubercular etiology independently predicted prolonged hospitalization.

Conclusion: Infective etiologies, particularly tuberculosis and parapneumonic infection, constituted the major causes of pediatric pleural effusion. Combined clinical, radiological, biochemical, and microbiological assessment significantly improved etiological diagnosis. Pleural fluid ADA demonstrated good diagnostic utility for tubercular pleural effusion. Early identification of complicated disease and timely intervention contributed to favorable clinical outcomes and low mortality.

## Introduction

Pleural effusion is the abnormal accumulation of fluid within the pleural cavity and reflects disruption of the normal balance between pleural fluid formation and lymphatic absorption. In children, pleural effusion is clinically important because it commonly indicates underlying pulmonary infection or systemic disease and may progress to respiratory compromise if diagnosis and treatment are delayed [1].

Pediatric pleural effusions differ from adult effusions because infective etiologies predominate. Pneumonia-related parapneumonic effusion and empyema remain major causes worldwide, while tuberculosis continues to contribute substantially to endemic regions such as India [2,3]. Delayed healthcare access, prior antibiotic exposure, malnutrition, and incomplete immunization may further increase the risk of complicated disease in resource-limited or semi-urban settings [3,4].

Clinical diagnosis is based on symptoms such as fever, cough, breathlessness, and chest pain and examination findings, including reduced air entry or decreased breath sounds. However, clinical findings alone cannot reliably differentiate uncomplicated effusion, empyema, tubercular pleural effusion, and non-infective causes. Therefore, a combined diagnostic approach using chest radiography, ultrasonography, hematological investigations, pleural fluid analysis, microbiological testing, adenosine deaminase (ADA) estimation, and cartridge-based nucleic acid amplification test (CBNAAT) is essential [5-7].

Pleural fluid ADA is widely used as a supportive marker for tubercular pleural effusion, particularly in high-burden settings where pediatric pleural tuberculosis is often paucibacillary and microbiological confirmation may be difficult [6,8]. Ultrasonography is useful for detecting loculation and septation and can guide pleural intervention. Microbiological tests, although often limited by prior antibiotic use, remain important for identifying bacterial pathogens and guiding antimicrobial treatment [4,9].

The clinical relevance of pediatric pleural effusion lies not only in diagnosis but also in its impact on management and outcomes. Children with loculated effusion, empyema, or persistent respiratory compromise may require intercostal drainage, intrapleural fibrinolysis, or surgery. Identifying etiological patterns and outcome predictors in a tertiary-care pediatric population can help improve early diagnosis, rational intervention, and clinical recovery. Also, there is a scarcity of such data in the current region. Therefore, the present study was undertaken to assess the clinico-etiological profile and outcome of pleural effusion in children admitted to a tertiary care hospital.

## Materials and methods

The study was a prospective observational study conducted in the pediatric ICU and ward of Dr. Susheela Tiwari Hospital, Government Medical College, Haldwani, a tertiary care center in Uttarakhand, India. The study population included all admitted patients with pleural effusion during the period of study. The study was conducted from 18 May 2024 to 20 November 2025.

Sample size

Eighty children were included in the study. The sample size was calculated with the following formula: $\mathrm{n} = \frac{4 \times \mathrm{P} \times \mathrm{Q}}{\mathrm{L}^{2}}$, where P = prevalence = 3% [10], Q = 97, and permissible error was taken as 4² = 16, giving n = 4 × 3 × 97 / 16 ≈ 80 after accounting for attrition.

Inclusion criteria

Children aged one month to 16 years and all patients with pleural effusion admitted to the pediatric ICU and ward in the pediatric department of Dr. Susheela Tiwari Hospital, Haldwani, were included in the study.

Exclusion criteria

Patients aged below one month or above 16 years and patients whose parents were not willing to give consent for participation in the study were excluded from the study.

Data collection

Detailed demographic data, clinical history, and physical examination were recorded. The following parameters were collected: tuberculin skin test, complete blood count, erythrocyte sedimentation rate (ESR), chest X-ray, serum total protein, serum lactate dehydrogenase (LDH), serum ADA, pleural fluid cell count, pleural fluid total protein, LDH, ADA, pleural fluid culture and CBNAAT, chest ultrasound, sputum Gram stain and culture, and blood culture and sensitivity.

Outcome assessment

Etiological diagnosis, management procedures, intercostal drain insertion, intrapleural fibrinolysis, surgery, complications, duration of hospital stay, prolonged hospital stay, and mortality were evaluated.

Case definition: tubercular pleural effusion (without microbiological confirmation via CBNAAT)

Patients met the diagnostic criteria if they presented with a consistent clinical history (e.g., persistent fever, cough, weight loss) along with a combination of the following supportive parameters.

Biochemical and Cytological Criteria

This included exudative pleural fluid exhibiting lymphocytic cellular predominance (found in 88.9% of tubercular cases) and elevated pleural fluid ADA >40 IU/L (found in 83.3% of tubercular cases).

Immunological and Epidemiological Criteria

The criteria included a positive Mantoux tuberculin skin test (reactive in 72.2% of tubercular cases) or a documented history of household contact with a tuberculosis patient (present in 27.8% of tubercular cases).

Radiological Criteria

Chest radiography or ultrasonography demonstrating pleural effusion alongside parenchymal pulmonary changes, hilar lymphadenopathy, or infective consolidations suggestive of tuberculosis (present in 77.8% of tubercular cases) were considered.

Exclusion of Bacterial Etiology

This comprised sterile pleural fluid bacterial cultures (present in 88.9% of tubercular cases) to rule out pyogenic empyema or simple parapneumonic effusion.

Ethics approval

The study was approved by the Institutional Ethics Committee, Government Medical College, Haldwani (861/GMC/IEC/2024/Reg. No. 824/IEC/R-22-04-204 dated 13.11.2024). Informed written consent was obtained from each patient's parent/guardian. The nature and consequences of the study were explained to them. Strict confidentiality was assured.

Statistical analysis

The collected data were recorded in Microsoft Excel sheets (Microsoft Corp., Redmond, WA, USA) and analyzed using appropriate statistical tests with IBM SPSS Statistics, version 20 (IBM Corp., Armonk, NY, USA). Quantitative variables were summarized as mean ± SD, while categorical variables were expressed as frequency and percentage. Logistic regression analysis was used to identify predictors of prolonged hospital stay, and receiver operating characteristic (ROC) curve analysis was used to assess the diagnostic utility of pleural fluid ADA for tubercular pleural effusion.

ADA cutoff criteria

The optimal cut-off threshold of 40 IU/L for pleural fluid ADA was determined using the Youden Index (J) derived from the ROC curve analysis; Youden Index (J) = Sensitivity + Specificity − 1; Using the diagnostic performance metrics at the 40 IU/L cut-off: Sensitivity = 83.3% = 0.833, Specificity = 77.3% = 0.773, =0.833+0.773−1=0.606 (or 60.6%) (Youden’s index). The threshold of 40 IU/L achieved the maximum vertical distance on the ROC curve.

## Results

This cross-sectional study was conducted on 80 pediatric patients diagnosed with pleural effusion over a period of 18 months. The largest age group was one to five years (28, 35.0%), followed by six to 10 years (24, 30.0%), 11 to 16 years (16, 20.0%), and one to 11 months (12, 15.0%). Males constituted 48 (60.0%) and females 32 (40.0%) of the study population. The head of the family was illiterate in 12 (15.0%); 20 (25.0%) had primary education, 28 (35.0%) had secondary education, and 20 (25.0%) were graduates. According to the Modified Kuppuswamy scale, four (5.0%) children were from the upper class, 16 (20.0%) from the upper-middle class, 28 (35.0%) from the lower-middle class, 24 (30.0%) from the upper-lower class, and eight (10.0%) from the lower class [11].

**Figure 1:**
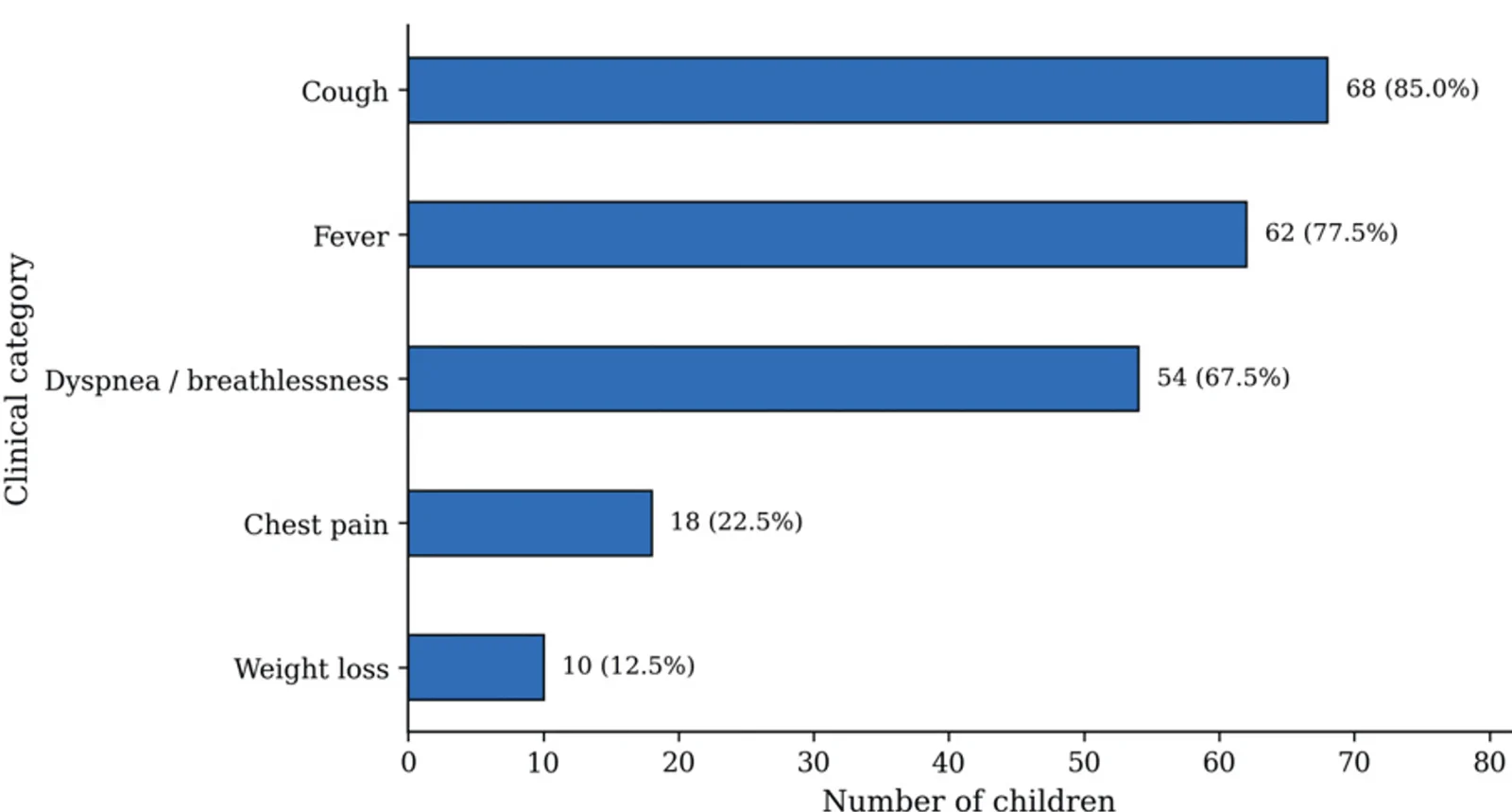
Presenting symptoms of the study participants (n = 80)

The chief presenting complaints were cough (68, 85%), fever (62, 77.5%), and dyspnea (54, 67.5%), followed by chest pain (18, 22.5%) and weight loss (10, 12.5%) (Figure 1). There was a history of recurrent respiratory illness in the past in 14 (17.5%) children; a history of tuberculosis contact with past recurrent respiratory illness was documented in 14 (17.5%) children; a history of tuberculosis contact in 10 (12.5%); and a documented pneumococcal vaccination in 22 (27.5%).

The Pediatric Asthma Severity Score (PASS), which assesses the work of breathing (score 0-2) and prolonged expiration (score 0-2), was used to grade the severity of respiratory distress on admission, with the maximum number of children showing moderate respiratory distress (44%, 55%) [12]. PASS score and examination findings are shown in Table 1.

**Table 1:** Pediatric Asthma Severity Score (PASS) severity and clinical status at admission among study participants (n = 80) Reference: [12]

Parameter	Category/Value	n (%)/Mean ± SD
PASS total score	Mild (0-2)	18 (22.5)
PASS total score	Moderate (3-4)	44 (55.0)
PASS total score	Severe (5-6)	18 (22.5)
General condition	Well	18 (22.5%)
General condition	Mildly ill	34 (42.5%)
General condition	Moderately ill	24 (30.0%)
General condition	Critically ill	4 (5.0%)
Heart rate	beats/min	118 ± 18
Respiratory rate	breaths/min	36 ± 10
SpO₂ on admission	%	92 ± 6
Fever >38°C on admission	Present	62 (77.5%)
Tachycardia	Present	46 (57.5%)
Tachypnea (WHO criteria)	Present	58 (72.5%)
Hypoxia (SpO₂ <92%)	Present	34 (42.5%)

**Figure 2:**
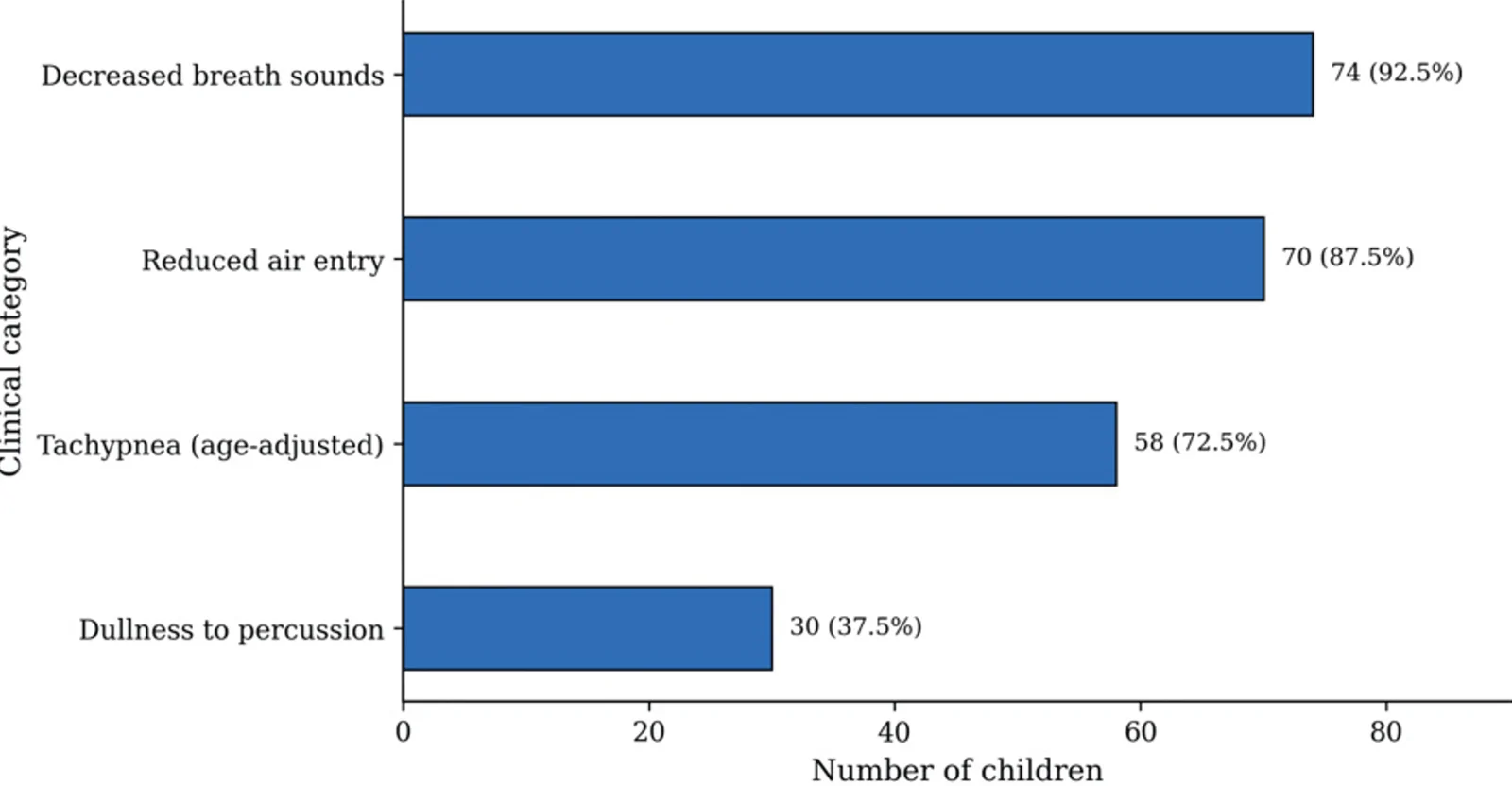
Clinical signs among study participants (n = 80)

Along with fever, tachycardia and tachypnea were most commonly noted in most children. About four (5%) children were critically ill with hypoxia, hypotension, and severe distress. Decreased breath sounds were the most common clinical sign observed (74, 92.5%), followed by reduced air entry (70, 87.5%), tachypnea (58, 72.5%), etc (Figure 2).

Radiological findings by X-ray and USG of the thorax showed a predominance of the right side (36, 45%) and non-loculated pleural effusion (54, 67.5%). Loculation in 26 (32.5%) indicated organized or complicated effusion (Table 2).

**Table 2:** Chest imaging findings on chest radiography and ultrasonography (n = 80)

Imaging findings	n (%)
Right-sided pleural effusion	36 (45.0)
Left-sided pleural effusion	20 (25.0)
Bilateral pleural effusion	24 (30.0)
Ultrasonography: free/non-loculated effusion	54 (67.5)
Ultrasonography: loculated/septated effusion	26 (32.5)

A diagnostic ultrasonographically aided pleural tap was done in all patients. The gross and biochemical characteristics showed it to be exudative in 68 (85%) patients and transudative in 12 (15%), as shown in Table 3.

**Table 3:** Gross and biochemical characteristics of pleural fluid among study participants (n = 80)

Parameter	Mean ± SD	Category	n (%)
Protein (g/dL)	4.8 ± 1.2	>3 g/dL	68 (85.0)
Lactate dehydrogenase (U/L)	750 ± 400	>1000 U/L	18 (22.5)
Adenosine deaminase (IU/L)	56 ± 34	>40 IU/L	52 (65.0)
Cell count	1600 ± 1100	Neutrophilic	34 (42.5)
Cell count		Lymphocytic	46 (57.5)
Effusion type	—	Exudative	68 (85.0)
Effusion type		Transudative	12 (15.0)

Pleural fluid culture was positive in 22.5% (18) cases only, with the predominant organism being *Staphylococcus aureus* (eight, 10%), *Streptococcus pneumoniae* (six, 7.5%), *Klebsiella *(two, 2.5%), and *Acinetobacter* (two, 2.5%), as shown in Table 4.

**Table 4:** Microbiological and molecular diagnostic profiles of the study participants (n = 80) CBNAAT: cartridge-based nucleic acid amplification test

Test/organism	n (%)
Pleural fluid culture positive (overall)	18 (22.5)
Staphylococcus aureus	8 (10.0)
Streptococcus pneumonia	6 (7.5)
*Klebsiella *spp.	2 (2.5)
Acinetobacter	2 (2.5%)
Pleural fluid CBNAAT positive	12 (15.0%)
Pleural fluid CBNAAT negative	68 (85.0%)
Blood culture positive	8 (10.0)
Blood culture negative	72 (90.0)
Methicillin-resistant *Staphylococcus aureus*	5 (6.25)
Escherichia coli	2 (2.5)
*Streptococcus pneumoniae* in blood culture	1 (1.25)
Mantoux reactivity present	32 (40.0)
Mantoux reactivity absent	48 (60.0)

**Figure 3:**
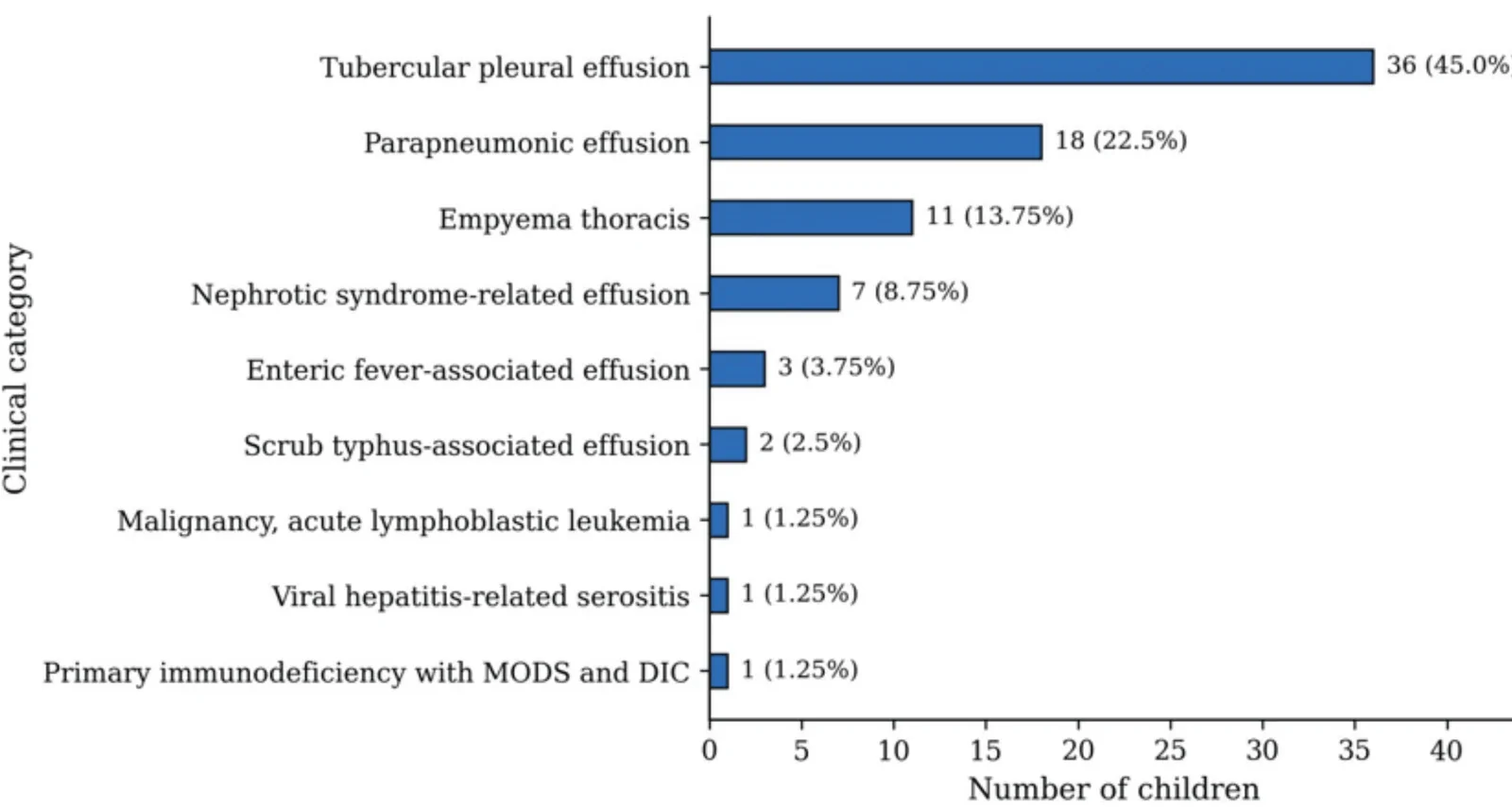
Etiological profile of pleural effusion in children admitted to the hospital MODS: multiple organ dysfunction syndrome; DIC: disseminated intravascular coagulation

Blood culture was positive in only eight (10%) cases, with predominant growth of methicillin-resistant *Staphylococcus aureus *(five, 6.25%), *Escherichia coli* (two, 2.5%), and *Streptococcus pneumoniae* (one, 1.25%). Tubercular pleural effusion was diagnosed in 36 (45%) children, with only 12 (33.33%) positive bacteriologically by CBNAAT and the rest clinically and radiologically. ADA > 40 IU/L showed a positivity in 32 (88.9%) children (Figure 3).

**Figure 4:**
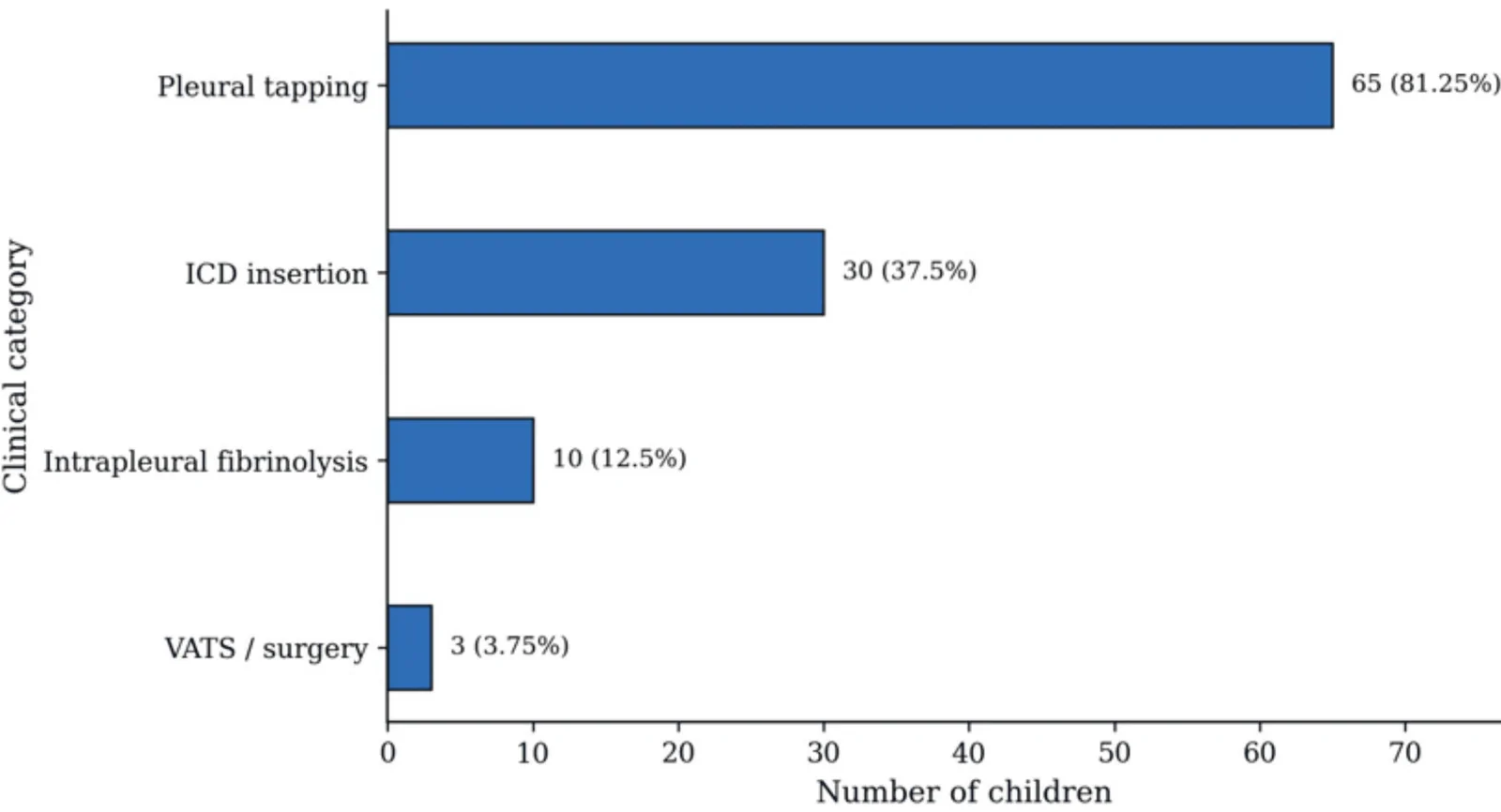
Management procedures performed among study participants (n = 80) ICD: intercostal drain; VATS: video-assisted thoracic surgery

Among 36 children with tubercular pleural effusion, 12 (33.3%) were microbiologically proven by pleural fluid CBNAAT, while 24 (66.7%) were diagnosed using a composite clinico-radiological and biochemical case definition without CBNAAT positivity. Tuberculosis contact was present in 10 (27.8%), straw-colored pleural fluid in 30 (83.3%), lymphocytic predominance in 32 (88.9%), pleural fluid ADA >40 IU/L in 30 (83.3%), Mantoux reactivity in 26 (72.2%), sterile pleural fluid bacterial culture in 32 (88.9%), and chest imaging suggestive of pleural effusion with parenchymal/infective changes in 28 (77.8%). Management procedures included pleural tapping (65, 81.25%), which was avoided in minimal effusion and those secondary to nephritic syndrome and a few synpneumonic effusions. Procedures were not mutually exclusive, and some children needed more than one intervention depending on response to treatment and clinical severity (Figure 4).

Table 5 shows the in-hospital outcomes, with a mean duration of 16 days. One mortality (1.25%) observed during the hospital stay was of a child with a submandibular abscess, multiorgan dysfunction with septicemia, and disseminated intravascular coagulation. Pleural effusion was a secondary complication. Complications were observed in 10 children (12.5%). Sepsis/ICU stay was documented in four children (40%), bronchopleural fistula in three (30%), pneumothorax in two (20%), and empyema requiring surgery in one child (10%).

**Table 5:** In-hospital outcomes among the study participants (n = 80)

Parameter	Value
Mean hospital stay (days)	16 ± 6
Prolonged stay (>7 days)	56 (70.0%)
Complications	10 (12.5%)
Mortality	1 (1.25%)

On multivariate analysis, ICD insertion, ultrasonographic loculation, and tubercular etiology emerged as independent predictors of prolonged hospital stay (Table 6).

**Table 6:** Multivariate logistic regression analysis of independent predictors of prolonged hospital stays (>7 days) ICD: intercostal drain; TLC: total leukocyte count

Variable	Adjusted OR	95% CI	p-value
ICD insertion	6.2	1.8-21.4	0.004
Ultrasonographic loculation	4.5	1.3-15.6	0.018
Tubercular etiology	3.1	1.1-9.0	0.036
TLC >15,000/mm³	2.2	0.7-7.0	0.18
Age >5 years	1.1	0.4-3.1	0.85

With CBNAAT positivity of only 36 (33.3%) cases, the present study finds ADA a useful test, with mean levels of ADA being significantly higher (84 ± 22) than non-tubercular effusion (43 ± 19), as shown in Table 7.

**Table 7:** Comparison of pleural fluid adenosine deaminase (ADA) levels in tubercular and non-tubercular pleural effusion (n=80)

Group	n	Pleural ADA (IU/L), Mean ± SD	p-value
Tubercular pleural effusion	36	84 ± 22	<0.001
Non-tubercular pleural effusion	44	43 ± 19	

**Figure 5:**
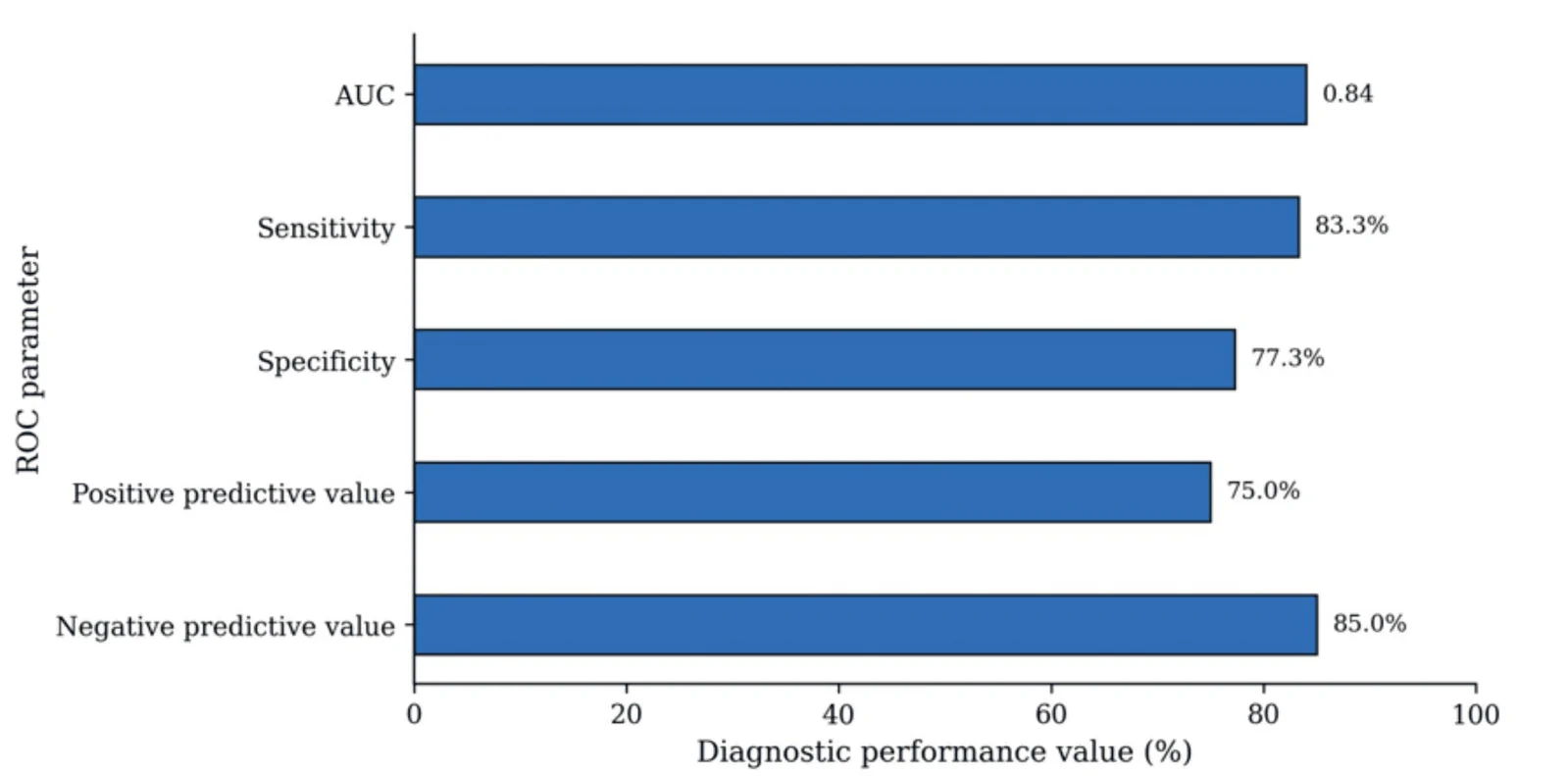
Diagnostic performance of pleural fluid adenosine deaminase (ADA) in identifying tubercular pleural effusion ROC: receiver operating characteristic; AUC: area under the curve

The ROC curve analysis showed an area under the curve (AUC) of 0.84 (95% CI: 0.75-0.92). At the optimal cut-off of 40 IU/L, sensitivity was 83.3%, specificity was 77.3%, positive predictive value was 75.0%, and negative predictive value was 85.0% (Figure 5).

## Discussion

The present study evaluated the clinical profile, etiological spectrum, and outcomes of pleural effusion in 80 children admitted to a tertiary-care pediatric setting. The largest proportion of cases occurred in the one-to-five-year age group, and males constituted 60.0% of the cohort. Similar pediatric studies have reported male predominance and higher burden in younger children, reflecting increased susceptibility to lower respiratory infections and infection-related pleural complications in early childhood [1, 3, 13].

Cough, fever, and dyspnea/breathlessness were the predominant presenting symptoms in the present study, while decreased breath sounds, reduced air entry, and tachypnea were the major clinical signs. These findings are consistent with Mehta et al., who reported fever, cough, and respiratory distress as common presenting complaints among hospitalized children with pleural effusion [14]. The symptom profile also aligns with reports from resource-limited and high-burden settings where infective pleural disease remains the dominant clinical pattern [2, 15].

Tubercular pleural effusion was the commonest etiology in the present study, accounting for 36 (45.0%) children, followed by parapneumonic effusion in 18 (22.5%) and empyema thoracis in 11 (13.75%). This differs from some Indian pediatric series in which parapneumonic effusion was the leading etiology, but it is consistent with the high regional burden of tuberculosis in North Indian and Himalayan populations [14, 16]. The high proportion of tubercular effusion in the present cohort may also reflect the tertiary-care referral pattern and the endemic nature of tuberculosis in the study region.

Pleural fluid analysis demonstrated a predominantly exudative pattern, observed in 68 (85.0%) children, with lymphocytic predominance in 46 (57.5%). Mean pleural fluid ADA was 56 ± 34 IU/L overall and was significantly higher in tubercular pleural effusion than in non-tubercular effusion (84 ± 22 IU/L vs. 43 ± 19 IU/L; p < 0.001). These findings support the role of ADA as an important adjunctive biomarker in pediatric tubercular pleural effusion as per National Tuberculosis Elimination Programme (NTEP) guidelines, similar to the findings of Shah and Gurnani and the systematic review by Na et al. [6, 8].

Microbiological yield was relatively low, with pleural fluid culture positivity in 18 (22.5%) cases and blood culture positivity in eight (10.0%). *Staphylococcus aureus* was the leading pleural fluid isolate, followed by *Streptococcus pneumoniae*. Low culture positivity is commonly reported in pediatric pleural infections, especially in children who receive antibiotics before referral or hospitalization [4, 9]. CBNAAT was positive in 12 (15.0%) children overall and confirmed 12 (33.3%) tubercular pleural effusion cases. This supports the known diagnostic role of CBNAAT as a specific but sensitivity-limited test in paucibacillary pleural tuberculosis [5, 7].

In terms of management, pleural tapping was performed in 65 (81.25%) children, ICD insertion in 30 (37.5%), intrapleural fibrinolysis in 10 (12.5%), and video-assisted thoracic surgery (VATS)/surgery in three (3.75%). These findings indicate that while most children required diagnostic or therapeutic pleural aspiration, a substantial subset had complicated effusions requiring drainage or advanced intervention. Similar pediatric empyema studies have highlighted the role of intercostal drainage and fibrinolytic therapy in improving pleural drainage and reducing progression to surgery [17, 18].

Outcome analysis showed a mean hospital stay of 16 ± 6 days, with prolonged stays beyond seven days in 56 (70.0%) children, complications in 10 (12.5%), and mortality in one (1.25%). In multivariate logistic regression, ICD insertion, ultrasonographic loculation, and tubercular etiology independently predicted prolonged hospital stay. These findings suggest that both disease complexity and underlying etiology determine hospital stays that indirectly reflect recovery duration. Loculated effusions and drainage-requiring disease reflect advanced inflammatory pathology, while tubercular effusion may require longer diagnostic confirmation and treatment stabilization.

Limitations

The main strength of the present study is the integrated assessment of clinical, radiological, biochemical, microbiological, molecular, and outcome parameters in a pediatric tertiary-care population. However, this was a single-center study with a modest sample size, and microbiological yield may have been affected by prior antibiotic exposure. Long-term post-discharge outcomes were not included, and rare non-infective etiologies were represented by small numbers. Despite these limitations, the study provides clinically relevant regional data on pediatric pleural effusion and its outcome predictors.

## Conclusions

The present study demonstrated that pediatric pleural effusion in this tertiary-care setting was predominantly infective in etiology, with tubercular pleural effusion being the most common cause, followed by parapneumonic effusion and empyema thoracis. Cough, fever, and respiratory distress were the major clinical symptoms, while decreased breath sounds, reduced air entry, and tachypnea were common examination findings. Most effusions were exudative, and pleural fluid ADA showed significant diagnostic utility for tubercular pleural effusion, with good ROC performance. Microbiological confirmation was limited but clinically useful where positive. ICD insertion, ultrasonographic loculation, and tubercular etiology independently predicted prolonged hospitalization. Early combined clinical, radiological, biochemical, and microbiological evaluation helped guide management and contributed to favorable outcomes with low mortality.
